# Supplementary material for: Epidemiology, injury pattern and outcome of older trauma patients: A 15-year study of level-I trauma centers
Source: PLoS One. 2023 Jan 30;18(1):e0280345. doi: 10.1371/journal.pone.0280345 (PMC9886263; doi:10.1371/journal.pone.0280345)
Supplement: S1 Table — Respiratory complications include acute respiratory distress syndrome; aspiration pneumonia; acute respiratory failure. Cardiovascular complications include cardiac arrest; myocardial infarction; pulmonary embolism; deep vein thrombosis; stroke. Surgery-related complications include postoperative haemorrhagic shock; abdominal compartment syndrome; anastomotic leak; evisceration/dehiscence. Infectious complications include: C.difficile colitis; catheter-related bloodstream infection; sepsis/severe sepsis/shock; wound infection; nosocomial pneumonia; osteomyelitis. Other complications include: acute kidney failure; decubitus ulcers; nonunion fracture; delirium; coagulopathy. (DOCX) [file pone.0280345.s001.docx]

**S1 Table: Evolution of complications over the years**

|  |  | 2003 | 2004 | 2005 | 2006 | 2007 | 2008 | 2009 | 2010 | 2011 | 2012 | 2013 | 2014 | 2015 | 2016 | 2017 | p-trend |
| --- | --- | --- | --- | --- | --- | --- | --- | --- | --- | --- | --- | --- | --- | --- | --- | --- | --- |
| Total, n (%) | ≥65 | 109 (10.2) | 158 (10.9) | 131 (8.4) | 292 (18.4) | 273 (17.5) | 280 (17.6) | 266 (17.2) | 337 (21.4) | 406 (24.0) | 399 (7.8) | 454 (27.5) | 526 (28.3) | 497 (26.6) | 531 (26.1) | 473 (22.6) | <0.001 |
|  | 16-64 | 100 (6.8) | 123 (6.4) | 87 (4.2) | 228 (11.1) | 196 (9.6) | 188 (9.0) | 182 (8.9) | 197 (9.8) | 205 (10.6) | 224 (11.6) | 252 (13.7) | 257 (14.6) | 288 (15.8) | 228 (13.5) | 272 (15.3) | <0.001 |
| Respiratory | ≥65 | 27 (2.5) | 47 (3.3) | 18 (1.2) | 35 (2.2) | 33 (2.1) | 40 (2.5) | 37 (2.4) | 46 (2.9) | 52 (3.1) | 51 (3.0) | 58 (3.5) | 79 (4.3) | 84 (4.5) | 94 (4.6) | 75 (3.6) | <0.001 |
|  | 16-64 | 36 (2.4) | 39 (2.0) | 20 (1.0) | 40 (2.0) | 41 (2.0) | 28 (1.3) | 24 (1.2) | 31 (1.5) | 38 (2.0) | 40 (2.1) | 58 (3.1) | 64 (3.6) | 58 (3.2) | 68 (4.0) | 62 (3.5) | <0.001 |
| Cardiovascular | ≥65 | 42 (3.9) | 60 (4.2) | 47 (3.0) | 93 (5.9) | 93 (5.9) | 82 (5.2) | 92 (5.9) | 99 (6.3) | 150 (8.9) | 115 (6.8) | 120 (7.3) | 142 (7.6) | 131 (7.0) | 119 (5.9) | 120 (5.7) | <0.001 |
|  | 16-64 | 34 (2.3) | 46 (2.4) | 34 (1.6) | 66 (3.2) | 73 (3.6) | 66 (3.2) | 61 (3.0) | 76 (3.8) | 73 (3.8) | 65 (3.4) | 77 (4.2) | 78 (4.4) | 90 (5.0) | 75 (4.4) | 104 (5.9) | <0.001 |
| Surgery | ≥65 | 5  (0.5) | 6  (0.4) | 4  (0.3) | 28 (1.8) | 21 (1.3) | 27 (1.7) | 24 (1.6) | 13 (0.8) | 26 (1.5) | 31 (1.8) | 44 (2.7) | 69 (3.7) | 49 (2.6) | 34 (1.7) | 22 (1.1) | <0.001 |
|  | 16-64 | 6  (0.4) | 13 (0.7) | 8  (0.4) | 24 (1.2) | 32 (1.6) | 26 (1.2) | 30 (1.5) | 36 (1.8) | 24 (1.2) | 20 (1.0) | 44 (2.4) | 38 (2.2) | 34 (1.9) | 35 (2.1) | 34 (1.9) | <0.001 |
| Infection | ≥65 | 4 (0.4) | 2 (0.1) | 4 (0.3) | 53 (3.3) | 42 (2.7) | 41 (2.6) | 52 (3.4) | 44 (2.8) | 40 (2.4) | 44 (2.8) | 51 (4.0) | 46 (2.5) | 57 (3.1) | 48 (2.4) | 39 (1.9) | <0.001 |
|  | 16-64 | 5  (0.3) | 7  (0.4) | 2  (0.1) | 90 (4.4) | 50 (2.5) | 62 (3.0) | 52 (2.5) | 47 (2.3) | 50 (2.6) | 49 (2.5) | 63 (3.4) | 57 (3.2) | 73 (4.0) | 62 (3.7) | 71 (4.0) | <0.001 |
| Other | ≥65 | 54 (5.1) | 72 (5.0) | 82 (5.3) | 184 (11.6) | 158 (10.1) | 162 (10.2) | 147 (9.5) | 231 (14.7) | 262 (15.5) | 277 (16.4) | 325 (19.7) | 391 (21.0) | 358 (19.1) | 396 (19.5) | 360 (17.2) | <0.001 |
|  | 16-64 | 38 (2.6) | 39 (2.0) | 40 (1.9) | 86 (4.2) | 88 (4.3) | 68 (3.3) | 79 (3.9) | 83 (4.1) | 94 (4.8) | 126 (6.5) | 131 (7.1) | 127 (7.2) | 136 (7.5) | 98 (5.8) | 142 (8.0) | <0.001 |

Respiratory complications include acute respiratory distress syndrome; aspiration pneumonia; acute respiratory failure

Cardiovascular complications include cardiac arrest; myocardial infarction; pulmonary embolism; deep vein thrombosis; stroke

Surgery-related complications include postoperative haemorrhagic shock; abdominal compartment syndrome; anastomotic leak; evisceration/dehiscence

Infectious complications include: C.difficile colitis; catheter-related bloodstream infection; sepsis/severe sepsis/shock; wound infection; nosocomial pneumonia; osteomyelitis

Other complications include: acute kidney failure; decubitus ulcers; nonunion fracture; delirium; coagulopathy
