# Supplementary material for: Epidemiology, injury pattern and outcome of older trauma patients: A 15-year study of level-I trauma centers
Source: PLoS One. 2023 Jan 30;18(1):e0280345. doi: 10.1371/journal.pone.0280345 (PMC9886263; doi:10.1371/journal.pone.0280345)
Supplement: S2 Table — (DOCX) [file pone.0280345.s002.docx]

**S2 Table. Missing data over the years**

|  | 2003  n=2,545 | 2004  n=3,378 | 2005  n=3,632 | 2006  n=3,642 | 2007  n=3,604 | 2008  n=3,685 | 2009  n=3,594 | 2010  n=3,589 | 2011  n=3,637 | 2012  n=3,617 | 2013  n=3,495 | 2014  n=3,623 | 2015  n=3,692 | 2016  n=3,723 | 2017  n=3,868 |
| --- | --- | --- | --- | --- | --- | --- | --- | --- | --- | --- | --- | --- | --- | --- | --- |
| Age | 0 (0) | 0 (0) | 0 (0) | 0 (0) | 0 (0) | 0 (0) | 0 (0) | 0 (0) | 0 (0) | 0 (0) | 0 (0) | 0 (0) | 0 (0) | 0 (0) | 0 (0) |
| Sex | 0 (0) | 0 (0) | 0 (0) | 0 (0) | 0 (0) | 0 (0) | 0 (0) | 0 (0) | 0 (0) | 0 (0) | 0 (0) | 0 (0) | 0 (0) | 0 (0) | 0 (0) |
| Mechanism | 0 (0) | 0 (0) | 0 (0) | 0 (0) | 0 (0) | 0 (0) | 0 (0) | 0 (0) | 0 (0) | 0 (0) | 0 (0) | 0 (0) | 0 (0) | 0 (0) | 0 (0) |
| Comorbidity | 296 (11.6) | 419 (12.4) | 399 (11) | 146 (4) | 368 (10.2) | 518 (14.1) | 570 (15.9) | 444 (12.4) | 312 (8.6) | 305 (8.4) | 168 (4.8) | 129 (3.6) | 135 (3.7) | 48 (1.3) | 178 (4.6) |
| ISS | 3 (0.1) | 0 (0) | 2 (0.1) | 2 (0.1) | 3 (0.1) | 6 (0.2) | 7 (0.2) | 4 (0.1) | 4 (0.1) | 4 (0.1) | 7 (0.2) | 2 (0.1) | 0 (0) | 0 (0) | 0 (0) |
| Discharge destination | 0 (0) | 0 (0) | 0 (0) | 0 (0) | 0 (0) | 0 (0) | 0 (0) | 0 (0) | 0 (0) | 0 (0) | 0 (0) | 0 (0) | 0 (0) | 0 (0) | 0 (0) |
| Bed-days | 40 (1.6) | 39 (1.2) | 64 (1.8) | 57 (1.6) | 57 (1.6) | 69 (1.9) | 77 (2.1) | 12 (0.3) | 0 (0) | 0 (0) | 0 (0) | 0 (0) | 1 (<0.1) | 0 (0) | 0 (0) |
| Mortality | 0 (0) | 0 (0) | 0 (0) | 0 (0) | 1 (<0.1) | 0 (0) | 0 (0) | 0 (0) | 0 (0) | 0 (0) | 0 (0) | 0 (0) | 1 (<0.1) | 0 (0) | 0 (0) |
