# Supplementary material for: Epidemiology, injury pattern and outcome of older trauma patients: A 15-year study of level-I trauma centers
Source: PLoS One. 2023 Jan 30;18(1):e0280345. doi: 10.1371/journal.pone.0280345 (PMC9886263; doi:10.1371/journal.pone.0280345)
Supplement: Attachment — Submitted filename: Response to Reviewers.docx [file pone.0280345.s003.docx]

December 6, 2022

Dr Yusuke Tsutsumi, academic editor

Plos One

Object: Submission of a revised manuscript

Dear Dr Yusuke Tsutsumi,

My co-authors and I would like to thank you for all your helpful comments regarding our manuscript entitled: " Epidemiology, injury pattern and outcome of older trauma patients: a 15-year study of level-I trauma centers". We believe the changes your reviewers suggested improve our manuscript's quality.

In the next few pages, you will find our point-by-point responses to your comments.

Please accept our sincere wishes, and we remain available for further inquiries.

Best regards,

Marcel Émond M.D. M.Sc. CCMF(EM) FRCPC

Clinician-scientist

Emergency physician, CHU de Québec – Hôpital de l’Enfant-Jésus

Associate Professor, Université Laval

1401, 18e rue

Québec (Québec) G1J 1Z4

Phone number: (418) 649-0252, ext. 66423

Fax number: (418) 649-5832

E-mail: [marcelemond1@me.com](mailto:marcelemond1@me.com)

**Journal requirements**

| 1. Please ensure that your manuscript meets PLOS ONE's style requirements, including those for file naming. The PLOS ONE style templates can be found at  <https://journals.plos.org/plosone/s/file?id=wjVg/PLOSOne_formatting_sample_main_body.pdf> and  <https://journals.plos.org/plosone/s/file?id=ba62/PLOSOne_formatting_sample_title_authors_affiliations.pdf> | Revised accordingly |
| --- | --- |
| 2. "AB received a scholarship from the Fondation du CHU de Québec (no grant number). This project was funded by the Fonds de recherche du Québec – Santé ( #33239). These funding agencies were in no way involved in the design, data collection and analyses of this study"  Please state what role the funders took in the study.  If the funders had no role, please state: "The funders had no role in study design, data collection and analysis, decision to publish, or preparation of the manuscript."  If this statement is not correct you must amend it as needed.  Please include this amended Role of Funder statement in your cover letter; we will change the online submission form on your behalf. | AB received a scholarship from the Fondation du CHU de Québec (no grant number). This project was funded by the Fonds de recherche du Québec – Santé (#33239). The funders had no role in study design, data collection and analysis, decision to publish, or preparation of the manuscript. |
| 3. We note that you have indicated that data from this study are available upon request. PLOS only allows data to be available upon request if there are legal or ethical restrictions on sharing data publicly. For more information on unacceptable data access restrictions, please see <http://journals.plos.org/plosone/s/data-availability#loc-unacceptable-data-access-restrictions>.  In your revised cover letter, please address the following prompts:  a) If there are ethical or legal restrictions on sharing a de-identified data set, please explain them in detail (e.g., data contain potentially sensitive information, data are owned by a third-party organization, etc.) and who has imposed them (e.g., an ethics committee). Please also provide contact information for a data access committee, ethics committee, or other institutional body to which data requests may be sent.  b) If there are no restrictions, please upload the minimal anonymized data set necessary to replicate your study findings as either Supporting Information files or to a stable, public repository and provide us with the relevant URLs, DOIs, or accession numbers. For a list of acceptable repositories, please see <http://journals.plos.org/plosone/s/data-availability#loc-recommended-repositories>.  We will update your Data Availability statement on your behalf to reflect the information you provide. | This study analyzed anonymous data from a provincial trauma registry (*Registre des traumatismes du Québec*). By provincial laws, the authors are not at liberty to share the datasets used for their analyses, as they were required to sign a non-disclosure agreement to access this data.  However, access to these datasets may be formally requested to the Commission d’Accès à l’information (<https://www.cai.gouv.qc.ca>) and the Direction de l’analyse et de la gestion de l’information of the Régie de l’assurance maladie du Québec (<https://numerique.banq.qc.ca/patrimoine/details/52327/49342>). |
| 4. Please include captions for your Supporting Information files at the end of your manuscript, and update any in-text citations to match accordingly. Please see our Supporting Information guidelines for more information: <http://journals.plos.org/plosone/s/supporting-information>. | Revised accordingly |

**Reviewer 1**

| Dear authors Thank you for giving me the opportunity to review this interesting manuscript. This theme is very important. Your manuscript was well written. So I have only one question.  Table 1; Why can you say there is significant (or non significant) change in Table1? Because you did not use statistical test in table 1. | No statistical test was previously performed to assess temporal changes. To improve the quality of the interpretation of results, we added a trend test. |
| --- | --- |

**Reviewer 2**

| 1) In the comparison between 2003 and 2017, it is stated that the mortality rate of the elderly decreased. However, referring to Fig. 4, there seems to be almost no change in the mortality over time in either group with ISS>12 or ISS≥25 (the authors might do a trend test). Comparing only the first and the last year and stating that mortality decreased is not well-founded, and it is recommended that the summary and conclusions be written in a less toned-down manner. | No statistical test was previously performed to assess in-hospital mortality temporal changes. To improve the quality of the interpretation of results, we added a trend test, as suggested. |
| --- | --- |
| 2) The percentage of severe lower extremity trauma in Tab. 3 for those 65 and older decreased significantly after 2012 and 2013. The discussion cites the improvement of osteoporosis and the contribution of a healthy lifestyle as the cause. However, it is unlikely to cause a marked decrease in severe trauma to the lower extremities after one year; the number of hospitalized patients aged 65 years or older from Fig. 1 increased sharply around 2014. It is recommended to consider other factors. For instance, was there a change in the local medical response, such as transferring severe lower extremity trauma patients to lower-level trauma centers? Is there a possibility of misclassification due to diagnostic methods and AIS calculations? | Thank you for this comment which allowed us to point out an error in this table. Absolute values were inverted between younger and older adults. This has been revised accordingly. Consequently, the decrease in the proportion of patients with severe lower extremities injuries is more regular over the years. |
| 3) Many observations included in complications are listed, but the aggregation method of these observations is unclear. Although the percentage of complications is shown in Fig. 6, a description of specific complications and characteristics could provide readers with helpful information for actual patient management. Although the amount of information would be enormous, consideration could be given to presenting only a few years or a few complications. | We have provided additional data about the type and the evolution of complications. This is depicted in S2 Table |
| 1) I recommend that the reasons for excluding deaths before or on arrival at the hospitals be described. | Given that we could not describe the injury mechanism or injury pattern in the group of patients deceased before or on arrival at the hospital, we decided to exclude these patients. This has been added in the method section.  Nevertheless, we wanted to investigate patient demographic characteristics, but we found that only 31 patients died before ED arrival and 57 on ED arrival. We believe that more patients may have died in one of these two periods but were not included in the RTQ, given that this registry aims to describe in-hospital trauma patients' pathway.  Hence, we believe that further investigation would not be of interest for readers. |
| 2) It may be helpful to specify the number of deaths before or on arrival at the hospitals. In that case, it is unlikely that a detailed search for the injury site would have been conducted, but if the age, rough cause of death, and trajectory of injury could be determined, it might be a helpful description. This is because this study aims to describe the epidemiology of patients transported to the trauma center, not to evaluate the treatment outcomes of the Level 1 trauma center. | See our response above |
